# Are Panoramic Images a Good Tool to Detect Calcified Carotid Atheroma? A Systematic Review

**DOI:** 10.3390/biology11111684

**Published:** 2022-11-21

**Authors:** María Prados-Privado, Javier García Villalón, Antonio Blázquez Torres, Carlos Hugo Martínez-Martínez, Juan Carlos Prados-Frutos, Carlos Ivorra

**Affiliations:** 1 Asisa Dental, Research Department, C/Juan Ignacio Luca de Tena, 12, 28027 Madrid, Spain; 2 Department of Signal Theory and Communications, Higher Polytechnic School, Universidad de Alcalá de Henares, Ctra. Madrid-Barcelona, Km. 33,600, 28805 Alcalá de Henares, Spain; 3 SysOnline, 30001 Murcia, Spain; 4 Faculty of Medicine, Universidad Complutense de Madrid, Plaza de Ramón y Cajal, S/N, 28040 Madrid, Spain; 5 Medical Specialties and Public Health Department, Faculty of Health Sciences, Rey Juan Carlos University, 28922 Madrid, Spain

**Keywords:** calcified carotid atheroma, panoramic image, risk factor

## Abstract

**Simple Summary:**

The goal of the present review is to investigate the reliability of panoramic dental images to detect calcified carotid atheroma. The findings of this systematic review exhibit that panoramic radiographs can be used for dental diagnosis and treatment planning, as well as to detect calcified carotid artery atheroma, so it can be a tool to prevent cardiovascular diseases.

**Abstract:**

To investigate the reliability of panoramic dental images to detect calcified carotid atheroma, electronic databases (PubMed, IEEE/Xplore and Embase) were searched. Outcomes included cerebrovascular disease events, cardiovascular disease events, patient previous diseases, and combined endpoints. Risk of bias was evaluated using the Newcastle-Ottawa Scale. Hence, 15 studies were selected from 507 potential manuscripts. Five studies had a low risk of bias, while the remaining nine studies were found to have a moderate risk. Heterogeneous results were obtained but showed that patients with risk factors, such as obesity, diabetes mellitus, hypertension, and smoking, and with calcified carotid atheroma on panoramic images, have a higher prevalence than healthy patients. The evidence in the literature was found to be equivocal. However, the findings of this systematic review exhibit that panoramic radiographs can be used for dental diagnosis and treatment planning, as well as to detect calcified carotid artery atheroma.

## 1. Introduction

The disease of the arteries characterized by the deposition of fatty material on its wall, forming atheroma plaques, is called atherosclerosis. These plaques may suffer calcification and project themselves into the vessel lumen, determining blood flow change [1].

Atherosclerosis is a disease that postpones its clinical manifestations and sometimes has no clinical symptoms. Therefore, any method to diagnose atheroma plaques is very important [2,3]

The most common images employed in daily dental practice are panoramic images. These kinds of images allow the professional to observe the area of the first cervical vertebrae, becoming an important tool to help in the early diagnosis of calcified atheroma in common carotid artery [1,4,5]. The calcified atheroma plaques can be detected in radiographies as irregular, circular, or heterogeneous radiopaque masses, unilateral or bilateral, which are usually located posterosuperiorly to the angle of mandible, approximately at the inferior margin of third cervical vertebra (between C3 and C4) near the hyoid bone [6].

The first to describe the presence of calcification by panoramic radiographs were Friedlander and Lande [7]. Several studies noted that by panoramic radiographs can be identified asymptomatic patients at risk for stroke [8,9].

The aim of the present review is to analyze the reliability of panoramic dental images to detect calcified carotid atheroma.

## 2. Materials and Methods

The present study employed the PRISMA (Preferred Reporting Items for Systematic reviews and Meta-analyses) statement to conduct and report the results [10].

### 2.1. Inclusion Criteria

The inclusion criteria were full manuscripts, including conference proceedings, that reported:

- Cohort and case-control studies evaluating the diseases associated with patients with carotid artery calcifications on panoramic dental radiographs.

### 2.2. Exclusion Criteria

The exclusion criteria were:

- Systematic or literature reviews
- No dental images application
- Editorials, commentaries, and letters to the editor
- Case reports, in vivo or cross-sectional studies that only report on the prevalence of disease.
- Studies that:
  o Did not assess the outcome(s) of interest
  o Did not analyze the prevalence of carotid artery calcifications with different diseases
  o Studies in which the patient suffered the adverse event before the dental radiograph was acquired
  o Evaluated calcifications outside the carotid arteries in the neck

There were no restrictions on the language or date of publication.

### 2.3. Outcomes

The consequences of interest were defined as:

(1) Cerebrovascular disease: stroke/cerebrovascular accidents, transient ischemic attack (TIA)
(2) Cardiovascular disease: angina, myocardial infarction (MI), heart failure
(3) Patient previous diseases
(4) Combined endpoints

### 2.4. Search Strategy

An electronic search was performed in the following databases up until 15 September 2022: MEDLINE/PubMed, Institute of Electrical and Electronics Engineers (IEEE) Xplore, and ScienceDirect. The search strategy used is detailed in Table 1.

### 2.5. Study Selection and Items Collected

M.P.-P. and J.G.-V. performed the bibliographic search and selected the articles that fulfilled the inclusion criteria. Both authors collected all the data from the selected articles in duplicate and independently of each other. Disagreements between the two authors were reviewed using the full text by a third author (C.I.) to make the final decision. The references of the articles included in this study were manually reviewed.

The following items were collected: authors, year, participant characteristics (sample size, gender, age), participants’ medical records, results of the study, and their main findings.

### 2.6. Study Quality Assessment

Modified Newcastle-Ottawa Scale was employed to evaluate the risk of bias of each study included in the present review [11]. Assessments were based on selection of study and control groups (4 stars), comparability of the groups (2 stars) and exposure, including outcome measurement and follow-up for at least 5 years (3 stars). The maximum possible quality score is 9 stars. Studies that scored 7–9 stars were considered as low risk of bias, 4–6 stars were considered as moderate risk, and 0–3 stars were interpreted as high risk of bias.

## 3. Results

### 3.1. Study Selection

**Figure 1:**
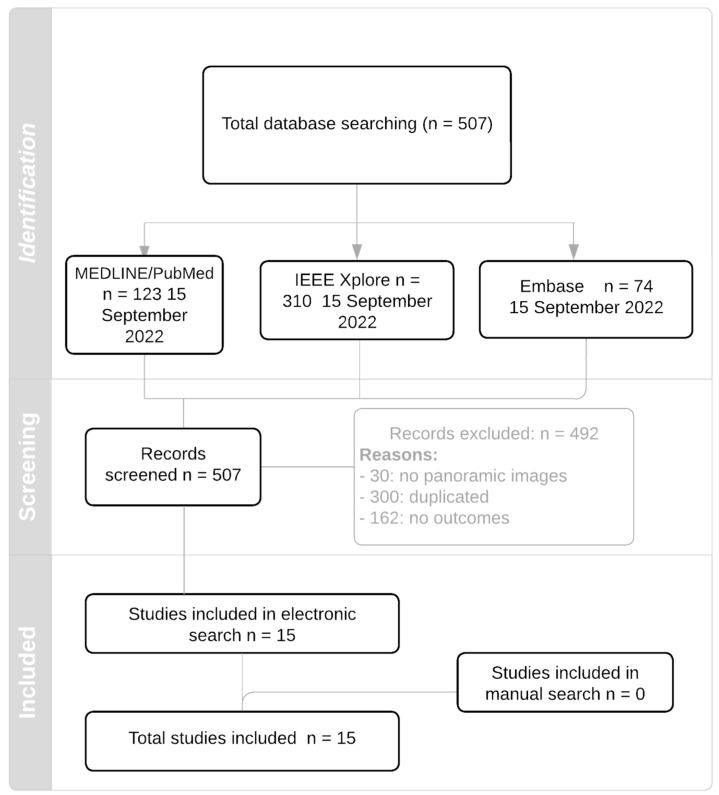
Flowchart.

A flowchart of how studies have been selected is detailed in Figure 1. 507 potential manuscripts were obtained among all of the electronic search strategies. A total of 492 studies were excluded due to they did not meet the inclusion criteria. Additionally, a manual search was carried out to analyze the references cited in 15 of the articles that were included in this work. Finally, no more articles were incorporated from the manual search. In the end, a total of 15 studies were analyzed.

### 3.2. Study Characteristics

The 15 included studies were published between 2002 and 2022. Two studies do not reported the age of the participants [12,13]. The remaining studies reported mean ages between 40 and 90 years.

Only four studies did not analyze hypertension on their patients [13,14,15,16]. Five studies analyzed smoking as a factor of CCA [13,14,17,18,19]. Two studies included dental diseases [16,20].

Only one study evaluated cerebral stroke/cerebrovascular accidents as an endpoint [8] and the conclusion achieved was that CCAAs often herald an ischaemic stroke. Four studies evaluated the influence of cardiovascular diseases in CCA [13,14,15,20].

The results are described in Table 2.

### 3.3. Quality Assessment

Table 3 details the risk of bias assessments. In view of the table, nine studies were found to have a moderate risk of bias and the other five studies had a low risk of bias.

## 4. Discussion

The aim of the present systematic review was to evaluate the reliability of panoramic dental images in detecting carotid artery calcifications (CACs). Friendlander was the first author to report, in 1981, the existence of soft tissue calcifications in panoramic images [7,27]. However, the questioning continues nowadays due to the conclusions of published studies in the field which evaluated whether carotid calcifications detected on panoramic dental images are associated with future events of stroke and/or ischemic heart disease. The conclusion was that the evidence of CCAs on panoramic images is related to an event of stroke and/or ischemic heart disease [28,29].

All studies included in the present review will be discussed in detail below.

Atalay et al. [17] determine the reliability of panoramic images as a tool to detect CCA by comparing it with Doppler ultrasonography examination. The study was carried out with 1650 patients older than 45 years. The participants were divided into two groups. Authors present a number of limitations. The most important is the need to clarify the clinical significance between panoramic images and Doppler ultrasonography, in addition to the analysis of the intensity, size, and shape of the calcification in the area of the carotid arteries on the panoramic images. The low number of participants in the study is also a limitation. Authors concluded that doctors must be aware of CCA on routine panoramic dental images, particularly in older patients.

Bayram et al. [14] evaluate the digital panoramic radiographs of 4106 patients older than 40 years for CAAs. Authors analyzed diabetes, diastolic blood, total cholesterol, smoking, and atherosclerosis, among other medical factors of participants, and concluded that the incidence of CCA was higher on the left side and that there were no statistically significant relationships between cardiovascular disease, coronary risk factors, and CAAs. Authors do not mention any limitation of their study.

Bueno Marinho et al. [20] investigate the prevalence of CCA on panoramic radiographs of 67 patients with liver cirrhosis and a mean age of 55 years. Authors concluded that cirrhotic patients are more likely to have CCA compared to healthy patients, and this risk increases significantly when kidney disease is involved. Authors do not mention any limitation of their study.

Chang et al. [21] analyzed if severity of obstructive sleep apnea is associated with the presence of CCA seen on 108 panoramic images. The study concluded that there is a strong association between severity of obstructive sleep apnea and the presence of CCA that can be seen on panoramic images. Authors mentioned two limitations: the small sample size and the use of a single center from where the data were collected.

Friedlander et al. [18] aimed to analyzed if the prevalence of atheromas is less in patients whose diabetes is noninsulin-treated compared with patients whose diabetes is insulin-treated. 46 neurologically asymptomatic patients (mean age 68.5 years) with type 2 diabetes were included in this study. Authors demonstrate that people with type 2 diabetes, irrespective of treatment modality, have high rates of atheromas. Authors do not mention any limitation of their study.

Friedlander et al. [22] evaluated the relationship between CCA on panoramic images and breast arterial calcifications on mammograms and concluded that there was not a relationship. A total of 40 women (mean age 62.2 years old) participated in the study.

Friedlander et al. [23] evaluated the presence of CCA on panoramic dental images on patient with primary hyperparathyroidism as a risk of factor of cardiovascular events on military veterans older than 50 years old. Authors concluded that CCA are often seen on the panoramic images of patients with primary hyperparathyroidism.

Friedlander et al. [24] studied the prevalence and severity of aortic arch calcifications on posterior-anterior chest radiographs women who also had CCAs visible on their panoramic images. This study concluded that prevalence of carotid plaque on panoramic images of women is significantly associated with presence of aortic arch calcifications on chest radiographs.

Friedlander et al. [25] assessed the possibility of CCA shedding emboli by observing their relationship with ipsilateral retinal emboli in males with diabetes, and the conclusion was that some male patients with diabetes mellitus type II having calcified carotid artery atheromas in the bifurcation area may have significant sequelae as evidenced by retinal artery emboli.

Friedlander et al. [12] determined the prevalence of calcified carotid artery atheromas in old men with gout. This study determined that CCAs often herald an ischaemic stroke in patients with gout, especially those with increased age, dyslipidaemia or diabetes.

Friedlander et al. [26] concluded that there is a strong association between the presence of CCAP seen on panoramic images of older males and extent of systemic inflammation as evidenced by elevated neutrophillymphocyte ratios.

Gustaffsson et al. [16] aimed to analyze the association between periodontitis and CCAAs among patients with a recent myocardial infarction and controls without a myocardial infarction and to evaluate whether CCAA combined with periodontitis is associated with myocardial infarction. The conclusion was that patients with periodontitis displayed CCAA in panoramic radiographs significantly more frequently than those without that dental disease, independent of whether the person had had a recent myocardial infarction. Participants with periodontitis combined with CCAA had a higher risk of having had a myocardial infarction than participants with either condition alone.

Ohba et al. [15] evaluated the incidence among 80-year-olds of CCAAs as detected on panoramic radiographs and concluded that panoramic images can be employed to detect CCA.

Patil et al. [19] evaluated the prevalence of calcified carotid artery atheromas detected on panoramic radiographs of patients with renal stones and assessed the correlation of renal stones and carotid artery calcifications. Their results concluded that there is no significant relationship between the presence of calcified carotid artery in the patients with renal stones and the control group.

## 5. Conclusions

The evidence in the literature was found to be equivocal. There are contradictory results regarding if there is or not a significant relationship between cardiovascular disease and CCA seen in panoramic images. However, the findings of this systematic review exhibits that panoramic radiographs can be used for dental diagnosis and treatment planning, as well as to detect calcified carotid artery atheromas. Therefore, panoramic images can be employed to alert clinicians and can provide potentially life-saving information.

Other risks of factors, such as age, diabetes, total cholesterol, obesity, diet, race, gender, or family history of cardiovascular diseases, must be evaluated to predict adverse vascular events. In view of the results achieved in the present review, diabetes mellitus, hypertension, and age should be the most important incident of strokes.

X-ray images must be analyzed carefully by practitioners in patients with risk, such as age, diabetes, or hypertension, in order to offer special indications.

## Figures and Tables

**Table 1:** Search strategy.

Database	Search Strategy	Search Data
MEDLINE/PubMed	atheroma OR carotid arteries OR calcifications AND (dental radiography OR dental image *) NOT review	15 September 2022
IEEE Xplore	atheroma OR carotid arteries OR calcifications AND (dental radiography OR dental image *) AND NOT review	15 September 2022
Embase	atheroma OR carotid arteries OR calcifications AND (dental radiography OR dental image *) NOT review	15 September 2022

**Table 2:** Descriptive characteristics of included studies.

Authors, Year	Participant Characteristics (Sample Size, Gender, Age)	Medical Records	Results	Main Findings
Atalay et al., 2015 [17]	Total n = 1650 (M 736/F 914) Age over 45 years. Group A (study group), CCA findings were confirmed by DU (n = 59); and Group B (control group), CCA findings were not confirmed by DU * (n = 34).	Hypertension Diabetes Smoking BMI (kg/m^2^)	- 5.63% were detected to have CCA on PR (M 43/F 50). Mean age of 59.84 ± 10.92 years. - No difference was determined in respect of CCA between the sexes (*p* = 0.745). - There was a significant difference between Group A and Group B in respect of hypertension (*p* = 0.004). - There was no difference between Group A and Group B in respect of age (*p* = 0.495), BMI (*p* = 0.756), diabetes (*p* = 0.168), and smoking (*p* = 0.482) distribution. - In Group A, these CCA findings were confirmed by DU imaging as hyperechoic signals with distal acoustic shadow. This examination showed 63.44% accuracy for the detection of CCA using PR.	Although PR cannot be used as an initial diagnostic method when searching for CCA, dentists should be aware of CCA on routine PR, particularly in older patients who may also have the risk factors of obesity, diabetes mellitus, hypertension, and smoking.
Bayram et al., 2006 [14]	Total n = 4106 (M 1678/F 2428). Age older than 40 years.	Family history Diabetes Systolic blood pressure Diastolic blood pressure Atherosclerosis Smoking, Menopause LDL HDL Total cholesterol	- CAA findings were detected in 88 patients (2.1%; 70 females, 18 male). - 50 patients had a radiopaque mass or masses on the left side, 20 on the right side and 18 on both sides. - 9 individuals were currently hypertensive. - 26.08% had a smoking history. - 4.34% had diabetes. - 21.74% had family history. 60.86% had elevated total serum cholesterol levels - 20% of the post-menopausal - women, of whom two (20%) had atherosclerotic plaque. - 30.4% had atherosclerotic - plaque that was not haemodynamically important, 4.3% had severe carotid artery stenosis and 65.3% had normal carotid artery.	- The incidence of CCA was higher on the left side. - There were no statistically significant relationships between cardiovascular disease, coronary risk factors and CAAs (*p* > 0.05).
Bueno Marinho et al., 2020 [20]	Total n = 134 (M 100/F 34). Mean age of 55 years old. 67 individuals with LC (case group) and 67 healthy individuals (control group),	portal hypertension, hypersplenism, hepatic encephalopathy, collateral circulation, spontaneous bacterial peritonitis, ascite, hepatorenal syndrome, hepatopulmonary syndrome, cirrhotic cardiopathy, coagulopathy, changes in red blood and white cells.	- Thirteen (19.4%) LC patients had CACA, whereas only four (5.9%) healthy patients had this condition. - LC patients are 3.72 times more likely to have CACA compared to healthy individuals (*p* = 0.02). - The presence of nephropathies increases the risk of development of atheromas by 18.58 times in cirrhotic individuals (*p* = 0.04)	- Cirrhotic patients are more likely to have CACA compared to healthy patients. - This risk increases significantly when kidney disease is involved. - It was also not possible to observe any statistically significant association between smoking and presence of calcified carotid atheromas
Chang et al., 2018 [21]	Total n = 108 (M 108/F 0) Mean age 54.7 ± 13.5 years old.	body mass index, diabetes, hypertension, hyperlipidemia and OSA	Approximately 1/3 of individuals (N = 33, 30.6%) presented with CCAP and this group was significantly older, and with greater odds of co-diagnosis of diabetes (*p* < 0.05).	- Strong association between severity of OSA and the presence of CCA
Friedlander et al., 2002 [18]	Total n = 58 (M 34/F 12) Mean age 68.5 years Patients with type 2 diabetes divided into two groups: NIT and IT	Hypertension, BMI, Smoking and HbA1c Level	- 24% of the NIT patients and 36% of the IT patients had atheromas; this difference was not statistically significant - The groups had similar risk factors: high levels of glycosylated hemoglobin A, or HbA1c; smoking; hypertension; and obesity (*p* > 0.05). - When compared with the 4% atheroma prevalence rate among healthy people of similar age, the rates were significantly higher in both the NIT (*p* = 0.02) and IT (*p* = 0.0006) patients.	- People with type 2 diabetes, irrespective of treatment modality, have high rates of atheromas
Friedlander et al., 2012 [22]	Total n = 40 women (mean age 62.2 ± 6.2 years old)	Ethnic, BMI, Hypertension, Diabetes mellitus, Dyslipidemia, Breast arterial calcification/mammogram	- A prevalence rate of 22.5% also had BAC. - The women with BAC tended to be older (65.1 vs. 61.3 years old), more frequently hypertensive (100% vs. 80.6%), and more frequently black than those without BAC, although these differences were not statistically significant (*p >* 0.10).	CCA of women is unrelated to the presence of BAC on mammograms
Friedlander et al., 2013 [23]	Total n = 60 (M 52/F 8) Patients older than 50 years old with PHPT	Hypertension, dyslipidaemia, diabetes and obesity.	- 40% had atheromas. - There were no significant differences between CCAP+ and CCAP− groups in gender or race (*p* > 0.05). - The atherogenic profile in the CCAP+ and CCAP− groups was not significantly different (*p* >0.05).	Calcified carotid artery atheromas are often seen on the panoramic images of patients with PHPT.
Friedlander et al., 2015 [24]	Total n = 72 (M 0/F 72) Older than 50 years One group (36) with CCAP and atherogenically risk-matched and another group (36) without CCAP.	Race, BMI, Hypertension, Diabetes mellitus, Dyslipidemia, Aortic Arch Calcification	- Women 60 years or older who had evidence of CCAP on their PR were significantly (*p* = 0.022; 95 percent confidence interval, 1.298–26.223) more likely to have evidence of AAC on their CRs than were similarly aged women who did not have evidence of CCAP. - This association was not evident in women younger than 60 years	Prevalence of carotid plaque on panoramic images of women 60 years or older is significantly associated with presence of aortic arch calcifications on CRs.
Friedlander et al., 2016 [25]	Total n = 100 (M 100/F 0) Study group: 50 neurologically and visually asymptomatic with diabetes, with CCAPs in PR and diabetic retinopathy in evaluation. Mean age of 66.6 ± 6.3 years Control group: 50 with diabetes who were matched for age and body mass index and had undergone both imaging studies and whose PIs were devoid of CCAP. Mean age of 66.1 ± 5.7 years old.	Diabetes mellitus, Diet control, Oral agent, Oral & insulin, Insulin only, Hypertension, Dyslipidemia, BMI	- The presence of asymptomatic retinal arteriolar emboli was found in the eye ipsilateral to the radiographically observed carotid atheroma in a 20% of the patients in the CCAP+ group, compared with 4% in the CCAP- group, and this difference was statistically significant (*p* < 0.03).	Some male patients with diabetes mellitus type II having calcified carotid artery atheromas in the bifurcation area, as visualized on PRs, may have significant sequelae as evidenced by retinal artery emboli
Friedlander et al., 2017 [8]	Total n = 531 with gout Group CCAP+: 163	Hypertension, Dyslipidemia, BMI, diabetes	- Logistic regression analysis demonstrated that a comorbid diagnosis of diabetes mellitus or dyslipidaemia, or advancing age was determinant in differentiating patients who were CCAA+ vs. those who were CCAA- (the panoramic image does not demonstrate a calcified carotid artery atheroma).	CCAAs often herald an ischaemic stroke and may be seen on the PIs of patients with gout, especially those with increased age, dyslipidaemia or diabetes
Friedlander et al., 2019 [26]	Total n = 100 (M 100/F 0) White men older than 55 years. Two groups of patients (n = 50 each) with plaque (CCAP+) and without plaque (CCAP–).	Hypertension, hyperlipidemia, and diabetes mellitus	- Group CCAP+ evidenced a mean NLR of 3.07 ± 1.43. - Group CCAP–evidenced a - mean NLR of 2.13 ± 0.68. *t*-test analysis comparison demonstrated a significant (*p* = 0.00007) - difference. - Logistic regression failed to show any significant relationship of NLR with the covariate/other variables of interest.	There is a strong association between the presence of CCAP seen on PRs of older non-Hispanic White males and extent of systemic inflammation as evidenced by elevated NLRs.
Gustaffsson et al., 2020 [16]	Total n = 1482 (M 1200/F 282) All patients have a PR that could be interpreted for the presence of CCAA. Study group: 738 (mean age 61.9) Control group: 744 (mean age 62.3)	Periodontally healthy, BMI, diabetes, University education	- There was no significant association between periodontitis assessed as bone loss and CCAA - The sex-stratified analyses of - cases disclosed an association between CCAA and periodontitis - among men (OR, 1.83; 95% CI, 1.28 to 2.64; *p* < 0.01) but not among women (OR, 1.04; 95% CI, 0.52 to 2.08; *p* = 0.90).	- Participants with combined periodontitis and CCAA had a higher risk of having had myocardial infarction as compared with participants with either condition alone. - CCAA and periodontitis were associated with myocardial infarction in an independent manner rather than an interactive manner.
Gustaffsson et al., 2022 [13]	Total n = 1482 (M 1200/F 282) Patients without previous MI and with available PRs were assessed for CCAA. Study group: 738 Control group: 744	diabetes, smoking status, treated and untreated systolic blood pressure, total cholesterol, and HDL cholesterol	- CCAA was detected on PRs in 206 control subjects (28%) and 251 patients (34%). - FRS was significantly associated with CCAA among control subjects (*p* = 0.04) and patients (*p* = 0.001). - SCORE was associated with CCAA among control subjects (*p* < 0.01) but not patients (*p* = 0.07). - Among men, FRS and SCORE were associated with CCAA in both control subjects and patients. - Diabetes was not significantly associated with CCAA after adjustments	Elevated cardiovascular risk scores were associated with CCAA on PRs among control subjects
Ohba et al., 2003 [15]	Total n = 659 (M 262/F 397) 80-year-old	body weight and height, physical stamina, blood pressure, electrocardiography, heel bone density, total blood cholesterol, and fasting blood sugar	- 5% of the patients were noted to have CCAAs. These appeared as a radiopaque nodular mass or masses adjacent to or just below the intervertebral space between C3 and C4. - There were marginally significant differences between males and females in CCAAs (*p* = 0.06). - 47% of CCAAs were detected in the right side. There appeared to be very little relationship between CCAAs and general and oral health.	Panoramic radiographs should be evaluated not only for pathosis of the teeth and jaws, but also, for other incidental findings, especially in the soft-tissue region of the neck
Patil et al., 2016 [19]	Total n = 240 (M 144/F 96) Study group = 120 Control group = 120 Patients with renal stones	Age, body mass index, high cholesterol, diabetes mellitus, hypertension, smoking, infarct	- A total of 25 (20.8%) patients with renal stones and 16 (12.3%) patients from the control group showed CAC. - The calcifications were higher in the patients with renal stones, but there was no statistically significant difference (*p* >0.05) between the two groups.	No significant relationship was found between the presence of CAC in the patients with renal stones and the control group. However, there was a trend for higher prevalence of CAC in renal stone patients.

* M = male; F = female; CCA = calcified carotid atheroma; DU = Doppler ultrasonography (DU) examination; PR = Panoramic radiograph; LDL = low density lipid; HDL = high density lipid; LC = liver cirrhosis; CACA = atheromas in carotid arteries; OSA = obstructive sleep apnea; NIT = noninsulin-treated; IT = insulin-treated; BAC = breast arterial calcifications; PHPT = Primary hyperparathyroidism; CR = chest radiographs; NLR = neutrophillymphocyte ratios; MI = myocardial infarction; FRS = Framingham Risk Score; SCORE = COronary Risk Evaluation.

**Table 3:** Risk of bias assessments.

Authors, Year	Selection (Maximum 4 Stars)	Comparability (Maximum 2 Stars)	Exposure (Maximum 3 Stars)	Total (Maximum 9 Stars)	
Atalay et al., 2015 [17]	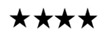	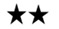	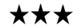	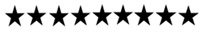	(9)
Bayram et al., 2006 [14]	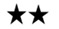	0	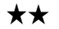	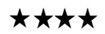	(4)
Bueno Marinho et al., 2020 [20]	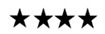	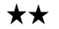	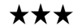	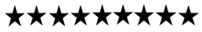	(9)
Chang et al., 2018 [21]	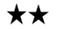	0	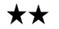	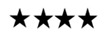	(4)
Friedlander et al., 2002 [22]	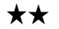	0	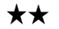	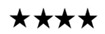	(4)
Friedlander et al., 2012 [23]	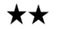	0	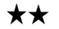	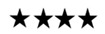	(4)
Friedlander et al., 2013 [24]	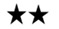	0	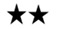	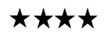	(4)
Friedlander et al., 2015 [20]	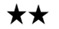	0	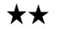	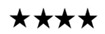	(4)
Friedlander et al., 2016 [25]	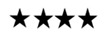	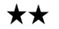	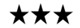	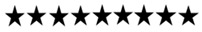	(9)
Friedlander et al., 2017 [8]	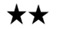	0	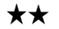	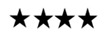	(4)
Friedlander et al., 2019 [26]	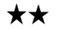	0	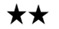	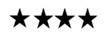	(4)
Gustaffsson et al., 2020 [16]	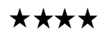	0	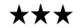	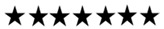	(7)
Gustaffsson et al., 2022 [13]	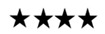	0	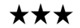	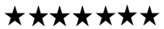	(7)
Ohba et al, 2003 [15]	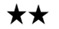	0	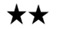	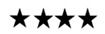	(4)
Patil et al, 2016 [19]	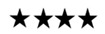	0	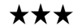	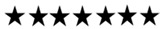	(7)

Rating scale: 7 to 9 stars = low risk of bias; 4 to 6 stars = moderate risk of bias; 0 to 3 stars = high risk of bias.

## Data Availability

Not applicable.
